# Virtual Daime: When Psychedelic Ritual Migrates Online

**DOI:** 10.3389/fpsyg.2022.819994

**Published:** 2022-04-29

**Authors:** Ido Hartogsohn

**Affiliations:** The Graduate Program in Science, Technology and Society, The Interdisciplinary Studies Unit, Bar Ilan University, Ramat Gan, Israel

**Keywords:** Santo Daime, psychedelics, entheogens, set and setting, online psychedelic ritual, online religion, online ritual, ayahuasca

## Abstract

During the 2020 COVID-19 epidemic a variety of social activities migrated online, including religious ceremonies and rituals. One such instance is the case of Santo Daime, a Brazilian rainforest religion that utilizes the hallucinogenic brew ayahuasca in its rituals. During the pandemic, multiple Santo Daime rituals involving the consumption of ayahuasca took place online, mediated through Zoom and other online platforms. The phenomenon is notable since the effects of hallucinogens are defined by context (set and setting) and Santo Daime rituals are habitually governed by punctilious regulations aimed at directing the experience of participants. The abrupt move to online space thus augurs significant implications in the context of hallucinogenic rituals. This paper looks at this development and its repercussions for entheogenic rituals, as it asks how do psychedelic rituals change when they move online? Building on the author’s previous work on set and setting in the Santo Daime religion, the paper introduces accounts from 12 semi-structured interviews with daimistas participating in online daime rituals, approached through the prism of set and setting, and the study of online religiosity. The analysis points at several key dynamics emerging in the context of virtual rituals. The migration online allowed for the continuation of Santo Daime entheogenic rituals at a time of social distancing, fostered a sense of global brotherhood and opened new possibility for religious participation and learning. Concurrently, online ritual produced an impoverished ritual experience and novel types of challenges including a higher potential for distractions, technical difficulties, and low sensory fidelity. Other novel challenges included social anxiety and an in-built tension between the social and spiritual dimensions of ritual. Finally, some participants were concerned by the cultural context of online rituals: technological mediation, consumerism, commodification, and digital divide. The limitations of digital technology appear amplified by the highly immersive, body oriented, experientially intensified context of the psychedelic experience. This paper contributes to the literature on the extra-pharmacological factors shaping experiences with psychedelics, as well as to the literature on the consequences of the adoption of digital media technologies during the COVID-19 pandemic.

## Introduction

The global eruption of the COVID-19 coronavirus pandemic in early 2020 has brought about a rapid wave of digitalization. With growing restrictions on assembly, diverse types of activities including shopping, learning, working, meeting and entertainment were moved online, a phenomenon that led to distinct challenges and adaptations (Dhawan, 2020; Okabe-Miyamoto et al., 2021; Vargo et al., 2021; Yan et al., 2021). A particularly interesting case in point is the story of the online rituals of the Santo Daime ayahuasca religion.

Santo Daime (henceforth referred to as SD) is a Brazilian rainforest religion that utilizes the hallucinogenic brew ayahuasca in its elaborate multiple-hour rituals that regularly evoke personal insight, communal intimacy and spiritual transformation (MacRae, 1992; Couto, 2004; Cemin, 2010; Blainey, 2021; Hartogsohn, 2021). As the COVID-19 pandemic spread across Brazil and the world in early 2020, SD churches were forced to cease their communal, on-site ritual service, with some of these churches convening on virtual platforms like Zoom. By May 2020 online SD rituals were rapidly institutionalizing. SD church leader, Alfredo Gregório de Melo (henceforth referred to by his common title Padrinho Alfredo), was leading virtual SD rituals of unprecedented scale, involving thousands of participants worldwide. These rituals, which were held under the banner of *Live Works*, raise a series of intriguing questions: What happens when entheogenic rituals migrate online? How are communal psychoactive rituals adapted to suit the pandemic reality of solitary, remote, computer-mediated participation? Which types of possibilities do online rituals open, and what types of challenges to they pose?

This paper can be read as a sequel paper to a previous one by the author that looked at the role of set and setting (context) in the shaping of Santo Daime entheogenic experience (Hartogsohn, 2021).^1^ The prior paper sought to provide a high-resolution description of the ways in which elements of ritual context (e.g., preparation, intention, visual, musical, social, and symbolic elements) shape the experiences of participants in SD Ceremonies. This sequel paper explores the transformation of the features of SD entheogenic ritual work as it migrates online, and how these transformations remold ritual dynamics and participant experiences.

As the mediatization of everyday life continues, a growing number of human activities migrate online (Hjarvard, 2013a,b; Couldry and Hepp, 2018; Hepp, 2019). The history of this process presents us with a Janus face. Digitalization and mediatization open new and liberating potentials (Turkle, 1997; Jenkins, 2006; Rushkoff, 2011) while also giving rise to experiences of loss, confusion, and despair (for prominent accounts see Carr, 2010, 2015; Turkle, 2011; Twenge, 2017a; Zuboff, 2019). As religious service, including entheogenic religious service, migrates online, its features and characteristics are reshaped, following the logic of mediatization. To understand the meaning of these new forms of digitally mediated religious life, it is crucial to keep a keen eye on the range of effects produced by the move online, and to tease out their implications for ritual dynamics, participant experiences, and religious institutions.

Santo Daime provides a particularly instructive case to explore since it employs elaborate ritual techniques that have been thoroughly described and analyzed elsewhere (MacRae, 1992; Cemin, 2010; Hartogsohn, 2021), and also because of the unique features of the psychedelic experience. Psychedelic compounds are distinguished by their tendency to amplify experiential intensity and enhance diverse features of sensory, emotional, and cognitive experience (Hartogsohn, 2018, 2020; Timmermann et al., 2020). The experience-enhancing effects of psychedelics serve to vividly highlight the characteristics, strengths, limitations and unique dynamics of online rituals.

Based on a comparison of the set and setting conditions of traditional SD rituals and their online equivalent, this paper draws on relevant insights from the field of media studies and on semi-structured interviews with 12 participants in *Live* works. It explores key features, tensions, and dimensions of online SD ritual, and sets out to answer the crucial question: can the ritual entheogenic experience be recreated online?

### A Brief Introduction to Santo Daime Religion

Santo Daime religion is a Brazilian ayahuasca religion, which emerged in the Amazonian state of Acre in the 1930s. Founded by Raimnudo Irineu Serra, SD rituals developed throughout the mid-twentieth century, until 1971 and the death of its founder (Moreira and MacRae, 2011). Following this event, the group split into several distinct lines. One of these lines, led by Sebastião Mota de Melo (henceforth referred to using his common title Padrinho Sebastião), went on to establish itself across Brazilian urban centers, and eventually became internationalized with centers in Europe, Asia, Australia, and the Americas.^2^ This paper concerns itself exclusively with Sebastião’s line, which achieved dominance over the past decades. Formally known as ICEFLU (Igreja do Culto Eclectico da Fluente Luz Universal), this line will henceforth be identified simply as Santo Daime (or SD).

Several key terms crucial to SD religion need to be introduced at this point. At the center of SD religion stands the ritual consumption of the ayahuasca beverage (referred to as *daime*) in a carefully organized context, centered around the joint musical performance of ritual hymns (*hinos*), within a ritual space referred to as the salon (*salão*). SD rituals are collective endeavors performed by congregations (churches) and referred to as works (*trabalhos*). During such works, members wear ritual uniform (*farda*) and believe to be receiving visions and guidance from the astral–a higher order spiritual realm, which becomes accessible under the force of daime. During works members believe to be creating a communal spiritual current (*corrente*) which binds participants together and elevates their spiritual work. They jointly face physical, mental, and spiritual hardships and traverse them by practicing firmness (*firmeza*) – a fortitude of spirit. SD works take place under the more general symbolic and religious framework referred to as “the doctrine” (*doctrina*), a “multi-vocal” term (Groisman and Sell, 1995, p. 250) invoked repeatedly despite never being properly defined, but generally pointing to the fundamental spiritual and metaphysical principles underlying and validating SD practice. Finally, a particularly important concept in terms of sociality and the introduction of the *Live* works is the concept of *Irmandade* (brotherhood). The SD irmandade consists of all members of the SD church. It portrays the members of the church as brothers and sisters, all parts of the ‘‘family of Juramidam.’’^3^ As we will see further on, the commitment to the preservation and cultivation of the *irmandade* played a key role in the decision to initiate online works.

### The Initiation of *Live* Works During the COVID-19 Pandemic

The year 2020 was supposed to be a festive, propitious year for the SD. For several years, the church has been preparing to celebrate the 100th year anniversary of its founding figure, Padrinho Sebastião (1920–1990), father of SD’s current spiritual leader, Padrinho Alfredo. Thousands of visitors from across the world were expected to travel to SD’s home community Céu do Mapiá in the Brazilian Amazon to commemorate the event in an unprecedented gathering of massive proportions branded as the *Centenario* (centenary).

The eruption of the 2020 COVID-19 Pandemic pulverized these plans. As the pandemic spread across Brazil and the world, international flight routes were suspended and Céu do Mapiá closed its gates to outside visitors. Meanwhile, SD churches worldwide began searching for solutions to the limitations put on SD religious practice by restrictions on congregation. As multiple religious congregations moved their services online (Campbell, 2020), SD churches too began organizing SD works using the Zoom videotelephony platform.

Santo Daime turn online was the result of dire religious exigency, a moment when religious communities worldwide were searching for ways of coming to terms with new restrictions on assembly (Lathrop, 2020). It posed distinct challenges. SD rituals are firmly grounded in the physical world and the possibilities it affords for immersive and immediate sensory experience, social copresence and interaction. The withdrawal from physical space was therefore potentially disruptive. It was nevertheless embraced by SD leader Padrinho Alfredo as a novel way of maintaining the SD *irmandade* and allowing its members to convene, sing, and pray together. Another factor which might have facilitated this openness to introducing digital media into the center of SD ritual might have been SD’s pronounced millenarian tendencies, reflected in the anticipation of a new era of great and unprecedented transformations (Orgad, 2012; Dawson, 2013). Like many other religious communities around the world, SD figures approached the COVID-19 pandemic as an event of religious significance (Dein, 2021). The insertion of digital tools into ritual work were thus readily understood by some as characteristic of an ominous time.

The first international live work headed by Padrinho Alfredo took place on the 26th of April, 2020. Crucially, the work chosen to start the *Live* era was a Saint Michael (Sao Miguel) work, a mediumship type work that was introduced into the doctrine by Padrinho Alfredo. The event was promoted across the entire worldwide SD brotherhood, many of which viewed this as an historic event demanding their recruitment. As one of my informants told me: “then came this invitation to do a *Live* work with Padrinho Alfredo (…) Doing a ceremony online wasn’t something I eagerly wanted to do, but this was an invitation from our Padrinho, and I felt like: ‘it’s not really a question’. We participate in whatever our Padrinho asks us.”

The ritual, which was organized by the NYC SD group Casa Cocar, was held on Zoom, and transmitted over Facebook Live and YouTube Live in collaboration with Canal Jagube, a Brazilian media organization involved in the dissemination of SD music and videos. According to the organizers, the event, which lasted 8 hours, attracted twenty thousand visitors, easily the biggest SD work in history (Canal Jagube, 2020a).

The months following that historical work saw a rapid succession of *Live* works. These events culminated in the Centenario celebrations which took place in October 2020. These events highlighted the *Lives’* potential to draw SD’s global community together. SD members were originally expected to travel to the Amazon to participate in the Centenario celebrations, yet most of SD’s global community would not have been able to make the costly and arduous trip. Through the *Live* works, an unlikely vision was realized: the entire global *irmandade* was suddenly able to participate together.

As *Live* works became a regular feature for global SD community their execution underwent rapid institutionalization. This institutionalization included the establishment of a core group of church members involved in the production of the *Live* works and their many technical, organizational, and promotional aspects, some of which are conducted in cooperation with Canal Jagube.

Online rituals can be divided into three major genres. The first and best known one, with which this article is primarily concerned, are the Live works organized by a dedicated group within ICEFLU. These official *Live* works take place on Zoom and are broadcast to two other platforms: Facebook Live and YouTube Live. They typically attract a viewership of several thousands.

Another type of zoom works is held by individual, local churches. In these smaller-scale rituals, which are not broadcast outside of zoom, church members are able to convene and consecrate the SD ritual beverage in the safety of their homes, without violating social distancing codes.

In addition to these, informants have also reported on hybrid type works where some church members convene to perform an on-site work, while others join them virtually, either for reasons of social distancing (for instance when a there is a cap on the number of participants allowed to convene due to COVID-19 restrictions), or because of being away from the country (For non-SD accounts of such models see Cocco and Bertran, 2021; Garner, 2021).

### The Ritual Status of Virtual Santo Daime Works

The question regarding the validity and authenticity of online rituals has stood at the center of much scholarly debate (O’Leary, 1996; Dawson, 2005; Helland, 2005). *Live* works have since their beginnings been surrounded by ambiguity. On the one hand, they have enabled the maintenance of community during calamitous times of social distancing and allowed the continued upholding of the SD calendar of spiritual works. Concurrently, the move online was met with the suspiciousness with which some in the global SD community approach modern technology.

The decision to reinvent the firmly established guidelines of Santo Daime ritual, honed over a period of almost a century, might have been contentious, yet, at a time of global pandemic, with the threat of social isolation and disruption to communal rituals, the choice was made, stimulated in part by local initiatives of Zoom-based daime rituals that began appearing as COVID-19 took hold. The considerable millenarian features of SD religion might have facilitated this transition. The COVID-19 pandemic, particularly in its beginning, carried biblical undertones, of a Godly reckoning and a fulfillment of ancient prophecies (Dein, 2021). To many of my informants, the switch online seemed to strangely make sense, perceivably becoming of the extraordinary nature of calamitous times. Still, despite this apparent embrace of the *Live* works, a certain ambiguity persisted. For one thing, it was never explicitly stated just what these works were or how they stood in relation to on-site works.

Theoretically, *Live* works can be considered entirely valid. If participants utter their opening and closing prayers and join in for the entirety of ritual, such works can be considered plainly as works at a distance. Practically, however, key differences emerge. Participants in *Live* works drink reduced amounts of daime (or, in most cases, none at all), are unbound from the ritual conventions enforced in on-site works and exhibit lower levels of commitment to ritual. Live works are therefore *not* the same as traditional, on-site works, as my informants confirmed. But if *Live* works are not “real” works, what are they then? Are they a temporary substitute? Do they aim to replace the ‘real’ thing? And if they are but a temporary substitute, will they persist in a hypothetical post-corona world? These are some of the questions this paper seeks to answer.

### On the Study of Online Religion and the Surprising Relationship Between Media Studies and the Study of Set and Setting

My study of online psychedelic rituals draws from two main bodies of knowledge. The first is the study of set and setting and their role in shaping psychedelic experiences, particularly in ritual context (Dobkin de Rios, 1984; Hartogsohn, 2017; Dupuis, 2021). The second is the field of media studies, in particular the media ecology approach which interrogates the effects on media on personal and social life (Strate, 2006, 2017; Cali, 2017).

The subject of set and setting and the field of media studies are closer abreast than one tends to suppose. This is readily perceivable when one appreciates that a crucial definition of the term ‘media’ is environment (Strate, 2017). Media ecologists have long approached the study of media as the study of environments, arguing that diverse media including writing, print, electronic media, and urban planning can be understood as environments shaping the features of human experience (McLuhan, 2003). Similarly, the study of set and setting is the study of (psychological, social and cultural) environments and their effects on shaping psychedelic experiences. Studying set and setting therefore means studying media (environment) in the context of psychedelic experimentation. Moreover, the study of the set and setting of computer-mediated, online rituals compatibly brings together these two seemingly disparate fields of study. It invites us to consider not only how media shape human interactions and experiences (the prerogative of media ecology) but also how our understanding of media effects might inform our analysis of the set and setting of online psychedelic rituals (a prerogative of psychedelic studies).

A key concept connecting the fields of media studies and the study of set and setting is the concept of affordance (Davis and Chouinard, 2016; Evans et al., 2017). Simply stated, affordances are defined as those possibilities which a certain medium renders available or unavailable. Affordances can be recognized each time an artifact requests, demands, allows, encourages, discourages, or refuses certain activities (Davis and Chouinard, 2016). While affordances may be fluid, non-binary, non-deterministic, and context-dependent, the concept does point to technological agency and the way structural features of a technology shape diverse aspects of the human world (Davis and Chouinard, 2016; Strate, 2017). Thus, for instance, the one-way, one-to-many affordances of television discourages the type of interactive, many-to-many, non-hierarchical public discourse facilitated by the affordances of digital social media, which in turn has profound implications for political and social life (Turner, 2006, 2019; Hopkins, 2016).

Migrations across media invariably lead to new constellations of affordances and constraints. The transition between on-site ritual work and computer-mediated ritual is no exception. As this paper demonstrates, migration online radically reconfigures entheogenic ritual dynamics. It opens a range of attractive affordances: the transmission of the work across time and space, the possibility for massive rituals, the recording, replay and re-experience of rituals. Yet, the new computer-mediated environment also entails new and at times uneasy constraints. Sound and video quality is inferior. Voice synchronization is impossible. Ritual leader is unable to survey his flock and offer individual support.

By thinking in terms of affordances we can better understand just what is gained and what is lost when SD entheogenic rituals migrate online. Affordances and constraints, in other words, are measures of set and setting, of opening and foreclosing psychic, social and spiritual landscapes.

This paper also converses with an additional field of scholarly investigation: the study of online religion. This field has been evolving since the 1990s and the rise of the internet, which became a formative force for contemporary religion (Højsgaard and Warburg, 2005; Campbell, 2011). Scholars of online religion have long been engaged in the study of online rituals and their relationship to embodied rituals (Grieve, 1995; O’Leary, 1996; Radde-Antweiler, 2006; Miczek, 2008). They have, among other things, developed theories of ritual transfer which argue that contextual changes to rituals (such as moving between media) lead to changes in their internal dynamics (Lüddeckens et al., 2005; Radde-Antweiler, 2006; Miczek, 2008), a fact amply demonstrated in the current study. Enlisting the findings of online religion scholarship to contextualize its results, this study also offers a unique contribution to this field by providing an examination of the distinct results of online ritual transfer in the case of psychedelics.

## Methodology

While participating as an observer in several *Live* works, I conducted in-depth semi-structured interviews (of between 30 and 60 min) with 12 daimistas from 6 churches in Europe, Asia, and Brazil, recruited partly through initial personal acquaintanceship and partly through further networking in a snowball manner.^4^ The interviewees had differing levels of experience with virtual daime works, with several who participated in a few dozen virtual SD works, and others who frequented only a few (often those voicing more critical views). The participant pool is not intended to be a statistically representative of the global SD community, but is rather used to portray, using personal testimonies, some of the significant issues arising through the online migration of SD ritual. The materials were analyzed for common themes, and against the literature on set and setting, media ecology, and online religiosity, as well as the author’s personal impressions and informal discussions with other visitors of these ritual, which all served as the basis for the examination.

## A Comparison of Set and Setting in On-Site vs. *Live* Santo Daime Works

The following sections build on my previous paper “Set and Setting in the Santo Daime.” What follows is a comparison of the set and setting of virtual SD rituals vs. traditional SD works, pointing to the fundamental ways in which the transition online alters the meaning of SD ritual.

## Preparation and Intention

The elements of preparation and setting of intention are essential for determining the quality of psychedelic experiences (Johnson et al., 2008; Danforth, 2009). In ‘‘Set and Setting in the Santo Daime’’ I pointed to key elements of preparation and intention setting in SD works including the perception of ritual order as divine in origin, the sanctified status of the daime beverage and the use of symbolism, and ritual calendar. Some of these elements remain unchanged (sanctified status of daime, symbolism). Other elements are modified by the move online. The divine origin of digitally mediated rituals, for instance, is not established as that of traditional SD ritual, a fact evident in the critical views expressed by some informants. The meaning of calendar is modified too, with users now able to access ritual recordings at any time.^5^

### The Paramount Role of Intention in the Home Environment

One point that recurred in many of my interviews is the paramount significance of personal preparation and intention in the context of a virtual rituals. Dawson, writing about SD ritual before the era of social distancing, observed: “collective ceremonial practice (…) furnishes a return on subjective cultic action far greater than that ordinarily available to an individual working in ritual isolation” (Dawson, 2013, p. 75). As ritual moves to a personal, isolated space, a massive loss of communal context results. Ritual participants are called forth to make up for these lost dimensions by preparing properly, adhering to ritual prescriptions, and cultivating a firm intention and commitment. Elements of proper preparation include, most prominently, putting on one’s ritual uniform (*farda*) and setting up an altar, like the one used in on-site SD works, including candles, flowers, water and a cruzeiro (caravaca cross). Importantly, in the beginning of each work a slide with the title “OFFICIAL PROTOCOL FOR PARTICIPATION IN ONLINE MEETINGS” is presented on the screen, wherein virtual participants are advised to follow certain guidelines: “Prepare the place with doctrinal references with a Cruzeiro and candle” “Stay in positions recommended in our rituals (sitting or standing), with appropriate clothing: blue or white farda, or white clothes” “Do not consecrate the Santo Daime or any medicine in front of the camera, during meetings.” (Canal Jagube, 2020b, min. 34:00). Such guidelines can be viewed as a drastically diluted and abbreviated version of ICEFLU’s “Norms of Ritual” document, which proscribes the proper execution of SD rituals, adjusted to the online environment (CEFLURIS, 1997; Hartogsohn, 2021).^6^ These guidelines gesture toward a proper manner of approaching virtual rituals. Yet, as several of my informants shared, commitment is often difficult to maintain due to the nature and constraints of the isolated home environment.^7^

A major component lacking from online ritual work and rendering it exceedingly more demanding to commit to is the absence of the communal observing gaze. In traditional works, daimistas sit/dance facing each other for the duration of ceremony. This creates an all-see-all, surveilling environment where a person is visible and potentially observed at all times, boosting participants’ level of ritual commitment. Daimistas will go to great lengths to avoid violating the rules and harmony of ritual space. Inspired by the SD’s spiritual-military dialect and the injunction to be loyal soldiers and hold the line during communal trials, participants may, for instance, resist bodily and mental impulses to lie down or visit the restroom, keeping themselves firmly seated even at times of great discomfort (Hartogsohn, 2021).

The solitary nature of online works often blurs such ritual boundaries, a fact that recurred in informant reports. Consider this report, by an informant that decided to join a long Zoom work but found that the home environment challenged her determination.

“I drank. I consecrated and I logged in […] About a hundred songs later I’m already at a different place. The phone keeps flickering, and I’m getting hungry. So I said to myself ‘Ok, I’ll go prepare something to eat’ and while I’m at it, I smoked a pito [rolled cannabis joint], and then looked at my phone. And then I said to myself ‘Okay, am I in a work or am I going log off?’ And you see this happening all the time. People log in and log off with their cameras. […] But then at some point I told myself: ‘wait a moment, but I’m sitting in this work now. So, what’s that about? Can I really do this just because I’m at home?’ where do you draw the line?”

Some informants found the freedom from the monitoring gaze liberating, allowing them to join in or leave the work as they pleased. Digital participants gain all sorts of liberties unavailable in on-site works: they may sit as they please, visit the bathroom, chat online, snack, or take a nap. The move online signals the end of the heteronomous order of SD ritual, where the daimista is subject to external control, and signals the recentralization of ritual around individual preferences. Radde-Antweiler proposes the concept of patchwork ritual, as ritual involving selection, transfer and combination of ritual elements (Radde-Antweiler, 2006). By allowing participants greater freedom, virtual SD works open the possibility of such patchwork rituals that cater to participant preferences in line with digital logics of customization and personalization of experience (Bennett, 2012; Kant, 2020).

At the same time, the new freedom presents new types of challenges. Participants face a growing number of choices regarding their level of participation: Which platform to use, what to wear, whether to open one’s camera, etc. This wider range of choices and optionality runs the risk of lowering satisfaction and inducing disquiet, as amply evidenced by the literature on choice overload (Schwartz, 2004; D’Angelo and Toma, 2017; Thai and Yuksel, 2017). Moreover, the spiritual benefit of SD works often arises from undergoing arduous trials. The strength to withstand such trials is often inspired by the social environment of the collective on-site work, where one is strongly encouraged to hold the line. As our informant found out, persisting in a work for an entire night is exceedingly more challenging when one is alone and unseen by others, highlighting the paramount importance of preparation and intention for those attempting virtual works.^8^

### Ritual Platformization and Its Consequences

A particularly interesting element of preparation and intention in virtual SD rituals is the relationship between the choice of media platform and participants’ level of engagement and commitment. One of my informants highlighted a distinction between joining in on massive *Live* works vs. small zoom works held by their local church.

“When I join a work by myself [*Live* work], I’m less observant about cruzeiro or farda. I care less. With the camera off nobody sees you. You can do what you want. But when I’m with my own church, even when I’m alone I’ll at least be wearing farda, and (…) everybody wears farda. There’s flowers, Candles. The whole shebang is there – to keep it as official as possible.”

My informants ascribed essential importance to the decision of joining works over Zoom vs. watching them on Facebook Live or YouTube Live. These three different media, all streaming similar content, furnish different types of affordances, leading to different types of experiences. Most crucially, while Zoom allows users to broadcast their own video stream and chat with other ritual participants, such possibilities are considerably more limited for YouTube Live and Facebook Live viewers, though a live comment option is available on both platforms, and was quite popular on YouTube during works. Helland (2000, 2005) drew a seminal distinction between the one-to-many, passive character of *Religion Online*, and the interactive, participatory character of what he terms *Online Religion*. Drawing on Helland’s distinction, which admittedly reveals a spectrum rather than a dichotomy (Helland, 2005), Zoom works are closer to the participatory *Online Religion* type in comparison with the more passive, viewership fostered by the Facebook and Youtube Live platforms.

Informants ascribed varying levels of spiritual merit to participation through different platforms. Connecting through Zoom was held in highest regard, while watching through YouTube or Facebook was considered second-rate. Importantly, though, both modes had their advantages and disadvantages:

“When I enter in zoom, I really enter. But if I play it on Facebook or YouTube, it’s like playing a hymnal. I can make dinner. I can be in bed. It’s just to watch.”

In the above quote the informant indicated the advantages of being able to join works through a secondary platform where they are not subject to ritual demands. The possibility to choose one’s level of engagement was viewed by some as a valuable affordance of digital space:

“You can choose your level of involvement. It’s not like going to a work. You can choose whether to watch on Youtube or enter the Zoom. When you enter the Zoom then you’re really participating.”

“You can be on Facebook, or peaking on Canal Jagube while you’re surfing [the web], or you can be on Zoom, but not open your camera. Stay in the background. Or on Zoom with open camera, or even performing. These are like different perspectives. Every position implies a different level of participation. […] Your choice of clothes will be in accordance with your level of commitment and motivation. You can either be watching Facebook wearing your pajamas, or you’re on Zoom, with trousers and a white shirt, or putting on the whole Farda.”

The ability to choose one’s level of involvement might be liberating. However, the liberatory affordances of digital media also presents a challenge. When participants opt for a lower level of exposure their commitment tends to be lower, and they may find it harder to engage with the communal current of the work. The solitary nature of digital works puts the onus on the participant to connect. One’s intention and commitment become paramount:

“You need to act. You need to participate on some level. That is fundamental to SD work in general. You can’t just come, drink daime and say ‘Well, what have you got to show me.’ If you want to work correctly, for your own sake, bring your energies either through singing or playing the maraca or other instruments. When I didn’t know anything, I’d tap my lap.”

The issues of preparation and intention are exceedingly acute in virtual SD works. Without the collective support of the *irmandade*, ritual participants are basically on their own. Without the presence of a ritual leader, or community, they can count only on themselves and their own intention setting. They are the ones in charge:

“It’s a very individual work. There’s no interference … So there’s no one else to whom you relegate responsibility. It’s all you yourself. You have nowhere to run.”

Asked about the challenges of *Live* works one of my observants summed this up the following way:

“The challenge is taking the work seriously, and concentrating to feel the force, receive the instructions, sing, and enter the current (…) the *Live* is an individual type of work of finding the firmness and the will to make yourself truly present independently of where you are (…) I believe that it depends on how each behaves during the *Lives*. If the person is connected and concentrated, drinking daime and singing, they are going to feel the force and receive the instructions in the same way as in the church. To my mind, it is a personal study of the force, of the will, and of firmness, for searching the divine within oneself.”

A final note on platformization, set and setting, and drug effects. As demonstrated above, choice of media has profound implications for participants’ experiences in virtual SD works. Informants viewed specific media platforms (Zoom) as more conducive to spiritual work than others (Facebook/YouTube). This view sits well with key insights from the literature on media ecology, which demonstrate that media types from writing to print and electronic media produce their distinct effects on the minds of users (Havelock and Havelock, 1963; Goody and Goody, 1977; Innis, 2007, 2008; Abram, 2012). Like drugs in the past, smartphones and social media apps have lately attracted scrutiny as potential catalysts and aggravators of mental pathologies (Twenge, 2017b; Haidt and Twenge, 2021). Considering the growing body of research interrogating the analogies, similarities, and interrelationships between media and drugs (MacDougall, 2012; Hartogsohn and Vudka, 2022), opens the intriguing possibility of conceiving of the interaction between drug use (ayahuasca and cannabis in the case of SD) and digital media use as linking two distinctly psychoactive levels – a pharmaceutic level and an electronic level, with the electronic level modulating the set and setting of the user, and therefore shaping pharmacological effects.

## Physical (Sensory) Setting

Physical (sensory) environment plays a crucial role in shaping experiences with psychedelics (Leary et al., 1964; Hartogsohn, 2015; Kaelen et al., 2018; Gukasyan and Nayak, 2021). The importance of sensory setting is largely a function of the heightened suggestibility characteristic of psychedelic states (Sjoberg and Hollister, 1965; Carhart-Harris et al., 2014; Hartogsohn, 2018). Within these highly suggestible states sights, sounds and scents are enhanced in meaning and may elicit positive experiences of absorption and epiphany next to harrowing experiences of anxiety and distress. Contemporary psychedelic therapy thus encourages the skilled use of visual elements and careful arrangement of musical selections (Johnson et al., 2008; Kaelen et al., 2018).

Santo Daime religion is meticulous in its management of sensory impressions (Hartogsohn, 2021). Prominent visual features of SD ritual space include the symmetric arrangement of participants in a hexagonal shape, at the center of which stands a star-table laid with candles, flowers, water, and the cruzeiro cross. This carefully arranged altar functions as a centripetal hub to which participants turn both physically and spiritually. Ritual space is habitually adorned with devotional objects such as the SD flag and religious icons. Members are dressed in festive uniforms (*farda*) (Dawson, 2013; Hartogsohn, 2021). The audial experience is defined by the musical performance of santo daime hymns, which guide the participants in their inner Journey (Labate et al., 2017). Olfactory dimensions, meanwhile, are engaged using incense and perfumes (Hartogsohn, 2021).

### On Disorder and Distractions in Domestic Space

Virtual SD works radically alter the established order of on-site SD rituals. Instead of a carefully arranged ritual environment, participants now find themselves at home, which entails a higher potential for encounters with the disorder and distractions endemic to domestic space. Rather than facing an altar and other ritual participants, participants now face a screen. An informant described the rearrangement of space in wistful terms:

“You invest all of your energies to make sure your screen is nice [to watch] and it means that instead of sitting in front of a photo of your padrinho or madrinha your photos actually face the cameras. (…) we’re used to sit in front of pictures of Master Irineu or Padrinho Sebastião or Madrinha Rita. And now we’re sitting in front of a screen. (…) it is like you’re worshiping a screen.”

Virtual ritual environment was described by informants as more distracting and social:

“You’re in front of a screen, usually a phone. There’s a lot of stuff [notifications appearing] that you don’t have in an official work, because you shut-off the background noise [of everyday life]. (…) There’s something social about it. Like using social media. It’s different than when you just meditate and concentrate and sing.”

The informant also shared a story of how her intention to participate in a work was thwarted by the pull of everyday life.

“*Dia das Mães* [mother’s day] was a work I really wanted to do. I set up the table and put the computer on it and started playing the guitar. But then in the middle, a friend came to visit, and it kind of cut my flow. It made me tired. And then after that break, I couldn’t come back. So I went to bed and opened it [the work] there and then I fell asleep.”

In this example, an event (a visit from a friend) which could not have occurred within the strictly regulated context of a church-based ritual subverted the informants’ plans to perform the work online. Several similar accounts were given by informants, who noted the difficulty of unlinking themselves from their web of digital and social relations when performing rituals from home.

### Sensory Fidelity

Participants in on-site entheogenic rituals take the high-fidelity of sensory impressions for granted. Physical space still reigns supreme in terms of richness of sensory experience. Some sensory dimensions, such as the olfactory impressions (e.g., from the burning of incense) or the kinetic, bodily experiences induced by lengthy dance works, vanish completely in virtual daime works. Other sense-organs are left radically impoverished. Popularly available screen- and streaming-based simulations of the human sensory experience pale next to immediate perception of sight and sound. Streaming is often choppy and disrupted. Some churches use inferior technical equipment producing pixelized images and grating sounds. In other moments uneven streaming causes peculiar and unsettling effects. The video and audio stream slow down or stop altogether, but then, as data finally arrive, the videostream speeds up to make up for lost time. The rhythmic disturbance may prove confusing and unnerving to those singing or playing at home.

“There are different types of technical problems. There’s sound drop [a faulty change of pitch]. Then there are gaps and getting stuck. … it can be annoying when buffering occurs. He [the guitarist] plays, then it gets stuck, then it plays the phrase super quick to connect [with the tempo], and this pulls you out of your natural current. Sometimes you’ll go on. Other times you’ll say. ‘Well, this is getting on my nerves. I don’t want it.”’

Whether or not the ritual participants are able to stomach such technical lapses, the quality of ritual unquestionably suffers. The transformational effects of SD rituals are based to no small effect on the possibility of surrendering oneself to the sublime beauty of ritual space, including the rich acoustics of live voices and musical instruments. The experience of sensory enhancement, common in psychedelic states, further enhances such pleasures. However, the choppy, low-bandwith, nature of online video and audio curbs the potential for such elevating sensory experiences and often proves unnerving.

Lack of voice synchronization is particularly detrimental to ritual dynamics. The poignant challenges of online religious singing have been priorly described (Miczek, 2008). In SD works, which are crucially musical (Labate and Pacheco, 2010; Labate et al., 2017), synchronization is a central element of ritual. By synchronizing their voices, participants become one voice. The paramount daimista concept of the current (*corrente*) is predicated on the notion that by meshing and synchronizing, human voices resonate to create a flowing, harmonious current. However, the built-in latency of the web’s architecture renders such synchronization impossible. This constraint of current virtual media has grave implications. As one informant told me: “Zoom has no possibility for synchronization. Instead of harmony, there’s separation. You don’t hear me, and you don’t hear the others. You just hear one source.”

### Transgressions of Space and Time

Electronic media is characterized by its ability to transcend boundaries of time and space. The attraction of virtual SD works lies, unquestionably, in their ability to transcend space-time and connect SD members separated by the COVID-19 pandemic and geographical remote locations. Concurrently, the transition to a space beyond space and time creates several unforeseeable, noteworthy effects.

A first point to mention is the increasingly dynamic, sometimes fleeting, nature of visual impressions in virtual SD rituals. In traditional SD works, one’s point of view is stable. One’s distance and angle in relation the altar-table and cruzeiro are fixed, the positions and identity of the people sitting or dancing at one’s side are generally immutable.

Virtual daime works offer a completely different experience. Here, a crucial part of the visual landscape appears not in the immediate physical space, but on the screen, in a dynamic videostream that moves around across different homes and churches. Hymnal works are commonly divided between churches in five-song sections, so that every five songs the participant is transported into a new visual and auditory environment. The pace of images rises rapidly in montage sequences. Here, the editing jumps around between participants’ video streams that are spotlighted for several seconds before moving on. Spacetime is thus condensed in a way that jogs the virtual participant across diverse landscapes with different features and characters. Crucially, these views are framed by the logics of mediatization (Hjarvard, 2008). The roving video stream moving between locations cannot but remind one of the new digital ecology of participatory culture (Jenkins, 2006), with its endemic selfies and endless live-streaming – of the burgeoning world of Facebook, YouTube, Tik Tok, Instagram and the aesthetics featured in web-inspired collective cinematic works like *Viral, In My Room*, and *Life in a day* (Bukowski et al., 2011; Bornstein et al., 2021).

A second point to note, is that the migration online transforms the implicit relationship between ritual space and spiritual benefit. In *Santo Daime: A New World Religion*, Andrew Dawson writes about a “Field of power” characterizing SD ritual space. Dawson’s “field of power” references a certain type of hierarchy where those in greater proximity to the physical center of SD ritual space are assumed to draw greater spiritual benefits in their ritual work.

Live works present a comparable but distinct hierarchy, reorganized according to the affordances of virtual space and the platformization of SD work. This hierarchy was described by one of my informants in the following manner.

“There are a couple of levels to the virtual astral. You can watch the *Live* the day after. It still counts. But if you are there live, that’s a different level (…) the next level is your camera is open. This is a much stronger connection. Then there is another level, which is when they spotlight your videostream on the big [main Zoom] screen. All your friends see you. Padrinho Alfredo sees you. Then there is another level, which is you appear on both video and audio [i.e., when performing].”

The informant distinguishes between five levels of ritual work, depending on timing and on one’s level of visibility. According to this logic, which recurred across the interviews, the more one reveals and is exposed on the videostream, the closer they are to the spiritual current of the work.

Another noteworthy way in which space-time are transgressed in virtual SD works concerns the use of pre-recorded performances. As noted above, hymnal works are commonly split into 5-hymn sections performed by different churches. Some of these sections are performed in real time, while others are pre-recorded, often for reasons of time difference (with non-Brazilian churches). These prerecorded sections are often a genre in themselves and are recorded under exceedingly diverse conditions. Participants might be under the influence of daime or not. They might record before, during or after an actual work. Alternatively, they might gather solely for the purpose of recording the short section.

Such considerations are highly consequential for the quality and “current” of the recording. Normally, different parts of works have different qualities, corresponding to their location in the temporal trajectory of the work. Human voices and musical performance sound different in different stages of the work, e.g., in the first hour vs. four or seven hours into a work, when participants are in a state of spiritual ecstasy, or conversely exhaustion and fatigue. These dynamics of traditional SD work are radically circumvented by the logics of pre-recorded performances, which are cut off from their habitual surrounding context, and where participant attempt to present their best selves to the watching audience.

“When we pre-record (…) there’s something very powerful about it, though a bit abrupt and weird. Sometimes you finish the recording in the force [feeling the force of daime], and then everybody goes their own way.”

The fragmented nature of time in online rituals has been noted by scholars (Grieve, 1995). One of my informants described the uncanny and sometimes disorienting experience of rewatching her pre-recorded video during a work:

“We shoot ourselves, then we send the video, and then they broadcast it and we watch ourselves live, but we can also watch it the next day, and watch ourselves watching ourselves. The work lives endlessly.”

Another informant who participated in a pre-recorded session excitedly shared their feelings of transcending time and space:

“It was very strong, because it’s still two days before the work, and you drink a bit and you sing, and you’re concentrated, and even though it’s pre-recorded you feel it very strong, and sometimes you look inside the camera, and inside the camera you come into contact with all the people who watch you, and Padrinho Alfredo and the comitiva, and Mapia and whoever is watching you. They are over there, inside the lens of the camera, and you can even wave at them, and you know that in a lag of time and space, they will see you (…) it just takes two days to travel to them. But you still waive at them, and two days later, you sit at home, and you watch it, and they wave back at you. You see them waive and you receive it.”

Another more mundane aspect of temporality involved in online works is the issue of time differences, which often make it more difficult for Daimistas from the other side of the ocean to participate in virtual works. It is quite different to enter a work that starts at 16:00 and ends at 23:00 (Mapiá time) than joining a work that starts at 23:00 and ends at 6:00 AM the next day (CET). Such time differences shape engagement levels.

“The hours are not easy, because if they start there in the afternoon here it’s very late. (…) This Saturday I was like ‘Well, I’m here with myself, and I had a really difficult day. I will not put my farda!’ Then I think: ‘but Padrinho Alfredo is there putting his farda. Well, if they see me without farda’ [that’s awkward]. But then I think: ‘for Padrinho Alfredo it’s 12 at noon, and here it’s the middle of the night. I have work. I have other stuff. I cannot be in white farda.”’

## Social Setting – Living in a Virtual Global Community

Sociality plays a crucial role in shaping experiences with psychedelics (Hartogsohn, 2015). Studies have demonstrated that social environment, levels of amenability and familiarity determine responses to psychedelics, and that sociality plays a key role in enhancing psychedelic effects (Dimascio and Klerman, 1960; Hyde, 1960; Olson et al., 2020). In “Set and setting in the Santo Daime” I discuss the central role of sociality in the SD experience, noting the central place of communitarian values in the SD doctrine, the “familial ideology” (MacRae, 1992, p. 3) embedded in SD culture, the socially cohesive effects of collective entheogenic ritual, and the tight-knit character of many SD communities (Hartogsohn, 2021). Importantly, a search for sociality has been noted as a primary driver toward online community (Grieve, 1995; Rheingold, 2000), and has played a central role in the turn online of many religious communities in the 2020 COVID-19 Pandemic (Campbell, 2020, 2021c; Cocco and Bertran, 2021).

### The Global Irmandade

The notably individual, isolated nature of virtual SD works was noted earlier [preparation and intention, section], and yet sociality is central to virtual SD works and might be considered their primary function. The desire to allow for assembly at a time of pandemic served as the original impetus for organizing *Live* works. Virtual works, despite their many limitations, were described as a way of keeping the irmandade close, and allowing SD members to convene, pray and sing together even at times of social distancing and isolation. Indeed, the one domain where virtual daime works undeniably surpass their on-site equivalent is their ability to bring together individuals from many parts of the world.

“How would people be able to do a work with so many individuals present as in the *Live*? People from Holland, India, Europe, Japan, Australia, United States. How would it be possible to unite all these people in a physical meeting? It would be difficult, don’t you think? (…) I’m not a person who is very connected to technology. (…) When I entered the *Live* I was very surprised, because I immediately understood the power of the *Live*. (…) It connected me with the *irmandade* of the entire world.”

The ability of virtual works to produce social connection on a global level was considered by informants as their most valuable feature. Some informants reported that participation in the works, reignited and strengthened their relationships with SD members from other churches. Others described the socially connective aspect of *Live* works as meaningful and uplifting.

“It’s good to know that as I start changing clothes [to ritual uniform], I know that another person in Europe is doing that, and another guy from Southern Brazil is doing the same, and then we meet in a way that we wouldn’t meet otherwise.”

“It’s nice because SD is such an international community and you suddenly have a chance to see people that you know from all over the world, and actually work with them.”

“When you share cyberspace with Padrinho Alfredo and other daime VIPs, that can give you a kind of feeling of closeness. It kind of elevates the session … and seeing people I know from other churches … The friendship warms the heart, and that can also be kind of spiritual.”

“These works connect the *irmandade* on a global level. You can be part of it wherever you are. They connect the *irmandade* with Céu do Mapiá. That was something new that we never could have imagined would happen, but it was a way for us to keep ourselves united, in prayer, singing the hymns and connected to the doctrine of SD.”

One of the features of online life is its ability to bring together sparsely spread communities sharing joint interests. For some of my informants being able to connect with Brazilian daimistas was valued as a way of finding peers and being able to transcend one’s sense of cultural marginality:

“We’re very esoteric in this country … here [online] you find many people that are esoteric together. They work with the same materials like me. In many ways they are closer to me than people in my country. … My everyday life is about daime hymns, so in this sense I’m closer to them [than to compatriots]. … There are many reasons why I don’t go to the [Amazon] forest. The heat, the humidity, mosquitos. Daime travels here. The only thing which doesn’t travel are Padrinho and his friends, and now we can be connected to them in this way and see them HD. … that’s an intimacy I wouldn’t have been able to achieve in Mapiá.”

An added value of global sociality was being able to celebrate the official dates of the full SD calendar, some of which are not regularly celebrated by smaller communities. “You use this opportunity because you want to commemorate the date, but you don’t want to drink alone. This then gives you a chance,” one informant noted.

Finally, for some, the participation in the global sociality of virtual SD works allows for spiritual reconnection to the Amazon Forest and its teachings, which are held in high esteem within SD religion.

“For people who were in the forest … I basically live with eternal *saudades da floresa* [yearing to the forest]. And [when watching] it’s like ‘Okay, it’s still there. It’s alive. They are there.’ It made me feel very connected. … It reinforces the connection to all the churches and the forest … Every time I saw the commitiva of Padrinho Alfredo, or the women in Mapia, or the people in Colonia 5,000 … Md. Rita, every time I saw these people that are so deep and central to the doctrine, I was reminded what *firmeza* is, and what essence these people bring to the works.”

### The Paradox of Virtual Daime

The central role of sociality in virtual SD works contrasts with the reduced role of another crucial element that traditionally stands at the center of SD works: powerful visions and inner voyages. Participants in virtual works tend to drink less daime. Mustering the courage to brave the ingestion of a second, third or fourth cup of daime is harder without the support of collective SD rituals. Furthermore, in on-site SD works experienced SD members (*fiscais*, guardians) are tasked with overseeing participants and helping them in need. Drinking heroic doses of daime is more daunting without this social safety net. There is, however, another reason why most of my informants found it difficult to surrender themselves to the entheogenic experience during *Live* works.

“Many times, the conclusion is that what I like better is getting together with a friend and doing a concentration work, just the two of us next to a candle, with a guitar. Because you can go deep. You can close your eyes. To go deep, it’s better to get together, or do it alone. But for the experience, the togetherness, the holiday, seeing other people – it’s [Live works] more in service of this need. … There’s a paradox, because really what’s the idea behind the Zoom? Is the idea having a shared experience or is it about going through a deep personal experience?”

The informant’s words are indicative of a greater issue at hand. An inherent paradox lies at the heart of the *Live* work which concerns the very idea of video streaming. Traditional SD rituals are habitually performed with closed eyes. SD members are often advised to keep their eyes closed during rituals to avoid intruding on other people’s experiences, and crucially, to connect more strongly with the force of daime and its visions.^9^ To go deep, one is advised to close their eyes and look inwards. However, when participating in a *Live* work it is only natural to open one’s eyes and stare at the screen. If not, after all, then why even open one’s laptop? The question reveals an unresolved tension between the spiritual and social dimensions of virtual SD rituals. The efficacy of one dimension reduces the efficacy of the other.

The dichotomy between the social elements of the work and the contradicting desire to go deep into the daime experience were evident in the words of another informant:

“When I am in a church, I close my eyes when I sing. But in the zoom works I don’t close my eyes. I look at the screen. If I open my camera, people can see me also. So I need to be prepared to be seen. … Usually I don’t like to be watched when it’s very strong.”

Does the social aspect of virtual daime works preclude the occurrence of deep spiritual experiences in the virtual rituals? While many of my informants found that sociality of *Live* work runs counter to their ability of deeply dive inward, experiences of spiritual elation and transformation *can* occur in the online contexts. Informants reported a range of spiritual experiences, usually induced by the social nature of works. While most informants felt that virtual rituals offered a diluted version of on-site SD works, in terms of their spiritual intensity, exceptions were noted. Particularly impressive was the report of one informant who said “when I sit down to do a zoom, I enter a church (…) I feel myself sitting on the bank next to the wall in the church.” The informant shared several spiritually uplifting experiences during *Live* rituals. One of these experiences that occurred in her first *Live* work helped her come to terms with the global situation of the pandemic and her own situation of social seclusion. Significantly, it was triggered by the social aspects of the *Live* work.

“I saw a woman alone. Another sitting next to an altar and a candle. One dancing. Another sitting. Another with a vase of flowers. A man alone playing the guitar. Another praying. It brought such a powerful emotion. It made me realize that each of us is firm with the words we sing like an ivory tower. And this will overcome whatever upheavals are coming. And this is what we’re doing during the pandemic: building that place where each can be. And this is the golden unity of these *Lives*. Because you’re sitting alone in your house drinking daime. [And you say to yourself] My god, what’s going to be? [Then] you see our Padrinhos, that which connects. You see the brothers. I saw the reunification of the web, of the earth, of the brothers with the spiritual web that people recognize when it brings people together.”

### Social Anxiety and Performativity

The above quote demonstrates the power of online sociality to induce spiritual experiences empowered both by psychedelics and the global affordances of virtual environments. At other points, though, the affordances and constraints of the digital social environment were experienced as oppressive and anxiety-provoking.

One distinct affordance of Zoom broadcasting is its unifocality. Unlike embodied SD works, where human voices mix together, and one is able freely move their gaze, here there is one spotlight, one videostream that is presented on the screen for all to see, focusing collective attention. The observing gaze thus becomes overtly noticeable, much like spectators viewing a broadcast performance. When spotlighted ritual participants come to resemble performers, this can have crucial implications. Stage fright is one of the most common human phobias (Narrow et al., 2002; Bodie, 2010). The prospect of suddenly appearing full screen in front of thousands of global viewers easily induces anxiety among many individuals. “It’s not easy to be watched by the whole doctrine [i.e., irmandade]” one of my informants told me. Social media has been implicated in enhanced anxiety (Vannucci et al., 2017). Importantly, in virtual SD rituals, the anxiety provoking effects of digital media may interact with the effects of other mind-altering plants that may aggravate anxiety, thus creating a second layer that reshapes the effects of the digital pharmakon (Hayatbakhsh et al., 2007; Hser et al., 2017). One of my informants, a guitarist, described his challenges knowing that his image and sound are broadcast to thousands.

“You’re exposed visually. How you look. Your guitar playing. I do these faces when I play the guitar. Suddenly all these twisted faces you’re making while you’re trying to reach F#, or something – Everybody sees that. It doesn’t matter that much in [traditional] works … When I view myself [on screen] there’s a whole range of emotions. A lot of self-critique about how I look, my guitar faces, every little thing. First, you’re overly self-conscious, then you realize it’s okay, not everybody are looking at you the same way you’re looking at yourself. I remember everybody [participating in a video recording] were anxious about it. Particularly when the video quality is high and the camera is directed at your face.”

While some agonize under the spotlight, others relish it. Next to social anxiety, another effect of the virtual unifocality is performance. The common element to both anxiety and performance is that both potentially distract ritual participants from the ethos of concentration in ritual. This is evident in montage sequences where the videostream roams between participant cameras. Spotlighted participants regularly recognize themselves on the screen. Some wave their hands at the camera, others bring their two hands together and gesture the heart sign. One of my informants found this type of performance anathema to daimista ethic:

“Even when you drink normal amounts [in *Live* works], something in the energy gets scattered. One obvious reason is people waving their hands […] or people doing the heart sign. Our comandante [church leader], forbids any waving. It’s less focused … It doesn’t have the kind of *firmeza* [firmness] that our doctrine requires.”

The informants’ remark against all types of waving and gesturing touches on a key point regarding the performative aspects of *Live* works. Participants in SD works are encouraged to close their eyes and explore themselves from within. One sings and plays musical instruments, but the emphasis is on being with the hymn, rather than on performance and self-consciousness. It is unthinkable for a daimista to wave their hands or gesture the heart sign during a traditional SD work. When *Live* participants wave their hands or gesture at the camera, they are in fact stepping out of their role as participants supporting the work through singing and concentrating. Instead, they are drawing attention away from the hymnal and toward themselves and inter-personal communication. Nevertheless, as lack of interaction can be interpreted as lack of engagement and care, such performative acts of waving and sending kisses to the camera are often encouraged. This type of behavior is therefore better tolerated because of the *Live* context where social function is central, though, as noted above, not everybody condones it.

Finally, one of my informants revolted against the amplified role of performance in its entirety, considering it to be blasphemous and opposed to the core of SD’s doctrinal teachings:

“You realize that you’re on a show. That you’re going to perform. I came to SD seeking for something that is not of the ego, that is not about performance, and not about showing myself to anybody. I felt like we’re in a movie, or a TV reality show. (…) the effort that we are used to put in our spiritual work is transformed into an effort that we put to show ourselves at our best.”

### Changes in the Irmandade

One final note on sociality. The rise of online SD works leads, among other things, to a shift in the constitution of the Santo Daime irmandade. As is evident from informants’ replies, daimista responses to virtual works vary. While some are open and welcoming to online rituals, others are more critical and less inclined to participate, because of ideological reasons, personal preferences, or technical limitations. SD members in areas under lockdown are more likely to join virtual works than those able to join on-site works. The make-up of participants in virtual SD works is therefore quite different from the make-up of traditional SD works. Compared to these traditional SD works some populations are excluded, other self-exclude, and other are over-represented. Since the initiation of *Live* works there emerged a distinct group of individuals regularly participating in these rituals. Virtual SD works therefore not only strengthen the connections within the *irmandade.* They also forge a new *irmandade* with an altered composition.

## Cultural Setting

The concept of cultural setting relates to impact of broad cultural forces on the character of psychedelic experiences (Hartogsohn, 2020). Wallace, for instance, argues that the Western association of hallucinations with mental illness raises the likelihood of negative experiences with hallucinogens for Westerners (Wallace, 1959). More recently, the concept has been used to explore the ways in which COVID-19, climate change, and political populism shape experiences with psychedelics for contemporary users (Prideaux, 2021).

The migration of SD works from the on-site church environment to platformized, digital environments entails a transformation of the cultural set and setting. One obvious way in which the cultural environment changes is the transition from the symbolically safeguarded environment of the church (perceived by daimistas to be protected by carefully arranged *puntos* with candles surrounding the church structure) into the suspect, exposed and perennially surveilled digital environment, mediated by corporate technology. On-site rituals are safeguarded not only by individual guardians taking care of the space and participants, but also by symbols and incantations. These elements are missing from online works, raising the question, how are virtual rituals protected?

The move online, implies that participant’s relationship with SD ritual is no longer immediate, but framed by digital media. Like many global citizens in the early 2020s, some in the contemporary global SD community are critical and suspicious of corporate dominated digital space, tarnished by association with massive online surveillance, rising inequality, voter manipulation, tech-addiction and mental pathologies (O’Neil, 2016; Alter, 2017; Bridle, 2018; Lanier, 2018; Eubanks, 2019; Zuboff, 2019). SD’s foundational story contains a strong primitivist message of return to nature and romantic simplicity. It tells of Padrinho Sebastião who, in the early 1980s, led his flock away from the Amazonian city Rio Branco and back to the depths of the forest. There, they were promised to find freedom and happiness in harmony with nature (Mortimer, 2018). The institutionalization of virtual SD works mars this narrative. The return to the forest ironically ends up leading back to the virtual. Instead of connecting directly with divine astral realm, participants (now users) must access it through the servers of disreputable tech corporations. Connection to divinity is now dependent on a proxy server and technological media, of which some participants are wary and distrustful:

“Sitting in front of the computer, I don’t like that. I sit all day in front of the computer. I don’t want my spiritual work to also be in front of the computer. This medium has the advantage that it can cross temporal and spatial boundaries, but it’s still sitting in front of the computer, with latency, and pixelization, and a quality [level] that has an unhealthy effect on my [somatic and mental] system. It can bring stress when things [in the videostream] get stuck.”

“I consider myself a millennial, so the world of commenting, reactions and live stories on Instagram is part of my world. But I don’t like to see a work transmitted on YouTube with all the comments … you see sides of daime people that you don’t really want to see.”

“The screen produces separation, which is something that’s not natural to us, considering the messages of the doctrine. Finding unity and connection in all of this is hard. We’re very used to the screen as something that turns us into spectators.”

Informants’ views of the virtual realm were often negative. One informant even referred to the digital dimensions as “the dark side of the astral.” Another informant argued that their participation in *Live* works is proof of their devotion to SD doctrine. They confessed to intensely disliking Zoom and video conferencing. To this informant, willingly participating in Zoom works meant that “this is a place I care so much about that I am willing to tolerate the medium, which I find difficult.”

The technological framing of virtual SD works also runs the risk of depreciating their value in the eyes of participants. As SD works become a consumable media experience, they run the risk of being commoditized and losing their aura effect. Walter Benjamin’s classic paper *The work of art in the age of mechanical reproduction* argues that the reproduction of artistic objects diminishes their aura effect (Benjamin, 2008). For example, whereas priorly one had to put on a frack and ride to the opera to listen to Beethoven, today one may listen to Beethoven on YouTube in the privacy of one’s home, wearing nothing but underwear. In this way, art loses its ritualistic resonance and potency. A sense of transcendence is lost with the change in context. A parallel can be found in the case of virtual SD rituals. Priorly, SD members needed to take an arduous pilgrimage to Céu do Mapia to participate in works with Padrinho Alfredo. Members’ commitment and struggle gave rise to reverence toward that which was reachable only with great difficulty and sacrifice. How might that change when one is able to frequent works led by Padrinho Alfredo on a regular basis, from the comfort of one’s own home? What changes when the *Live* stream of a spiritual leader appears next to other videos placed there by Facebook’s and YouTube’s algorithms, some of them blatantly inappropriate to religious service? In sum, how do the flattening, de-hierarchal features of digital technologies (Castells, 2003; Turner, 2006) change participants’ modes of approaching religious authorities and do they corroborate reports of erosion of religious authority in digital environments? (Piff and Warburg, 2005; Hjarvard, 2008; Cheong et al., 2011). Some of my informants’ reports imply that the *Live* context may introduce acrid alterations in one’s relationship with their spiritual guide and spiritual work.

“It’s like the gentrification of the doctrine. There’s this Hollywood vibe. I want my Padrinho looking me in the eyes and serving me daime, not somewhere on a screen. It creates *rebeldia* (rebelliousness) in me”

‘‘SD is becoming something you consume. You have products to consume. You have webinarios [webinars]. You have recordings. You have merchandise of SD.’’^10^

A related issue that came up was the culturally delicate issue of class differences. The question of digital divide has cropped up in recent discussions on the virtual turn in online religiosity (Campbell, 2021b). Such differences may play a role in on-site SD works, as evident when comparing the material conditions of SD congregations: the architecture of SD churches, the use of makeshift vs. permanent constructions, the fee paid for participation and the type of ayahuasca procured. Still, while important on a macro level, such factors tend to be subdued within the person to person level of an on-site work, where participants operate within the same conditions (occupying the same space, using the same equipment, drinking the same beverage, etc.). In online works, where participants exist and transmit from distinct types of environments, such differences become more apparent, as reflected by the quality of one’s technical equipment and conditions: internet connection, speakers, screen, and personal space. As one of my informants argued:

“In the doctrine we are ‘todos iguais’ [all equal] and you can give in to a sense of oneness, but technologically we’re not all equal. If I have a Mac and I’m sitting in my penthouse connected by an optic fiber, and you have a small apartment with a sleeping baby then we’re not ‘todos iguais’. There are class differences, and this is in a sense a privatization of the Salão.”

Finally, another form of commodification regards the concern that the logics of SD work might become subordinate to the logics of digital consumption. Writing about the mediatization of religion, Hjaravard observes that “through the process of mediatization, religion is increasingly being subsumed under the logic of the media” (Hjarvard, 2008, p. 11). In the case of SD online ritual, by entering the video player ecology, the spiritual arguably becomes manipulable. The viewer (formerly participant) may opt to skip certain less compelling sections of the work (e.g., lengthy prayers, breaks, concentration sessions), rewatch highlights or fast forward through other sections. Religious ritual is transformed from lofty and awe-inspiring to a pliant, manipulable media object which users can use as they like, and even binge on.

## Integration

Integration is the post-session work of constructively processing and assimilating the experiences and insights that arose during a psychedelic experience (Godasi, 2019; Westrum and Dufrechou, 2019; Watts and Luoma, 2020). In on-site SD rituals, informal integration habitually takes place after the work. Participants often linger in ritual space exchanging hugs, impressions, and engaging in reinterpretations of their experiences. This dimension is lost with the migration online. While Zoom participants are able to remain in the Zoom room as long as it is open, the possibility to freely converse is limited by the unifocality of the Zoom platform: the inability to have discrete conversations without being heard by all. For *Live* viewers, even this is impossible. Once the *Live* videostream ends, participants find themselves alone in their home environments. Participating in a *Live* work as a group may mitigate the sudden cutoff and allow participants to integrate with their close group, as noted in footnote no. 8.

## Skillset

The concept of skillset is defined as a set of cultivable techniques, strategies and approaches for navigating experiences with psychedelics (Godasi, 2019). On-site SD rituals offer participants diverse opportunities to cultivate skillset including studying hymns, practicing concentration, and fulfilling diverse ritual roles such as playing musical instruments, acting as guardians, or serving daime. Each of these roles offers distinct challenges, curricula and skills to be perfected (Hartogsohn, 2021). In virtual SD works, however, many of these dimensions are lost, as one informant argued.

“In our rituals we have alter egos that emerge, and people can access powers that are not necessarily available to them in their everyday life. … The relationships between the brothers and sisters are powerful because we tend to produce some other versions of ourselves during the work, which we do not do on zoom.”

Because of the affordances of video transmission (i.e., the unifocality of video and audio streams), and because there is no common ritual space, participants’ ability to contribute to the collective ritual (e.g., by singing, dancing, or facilitating other aspects of ritual) are limited. This changes the nature of skillset, which is achieved not in communal work, but in solitary work, developing the ability to stay firm, intent, and concentrated away from the supportive networks of social ritual work.

Another dimension of skillset that may develop in virtual SD works is the pedagogic deepening of knowledge and familiarity with SD’s hymnal repertoire and manners of ritual. By participating in works led by SD dignitaries of international renown, participants may learn from others, more knowledgeable in the SD work:

“It’s an education. It’s learning the hymns, the musicality, the energy … you see how other churches play, their way of working with different musical instruments … with the singers … there are many aspects to this.”

## Can Virtual Santo Daime Works Replace On-Site Works?

A central question raised in this paper, regards the future of *Live* works in a post-pandemic world. As noted, the COVID-19 pandemic has led to a rapid wave of digitalization in diverse areas of life, including business, education, culture, and therapy. In many cases, digitalization has been presented as irreversible. The world of business, education, and culture, some argue, will never revert back to ‘normal.’ Similarly, in the ecclesial domain, some argue that the church cannot return to the life and practice of pre-pandemic days (Campbell, 2021b; Garner, 2021; Rieimann, 2021). Can the same be said regarding with entheogenic rituals?

The challenges of translating religious rituals into online environments have been noted by early scholars of online religiosity, some of whom opined that current internet technology will struggle to replace ritual performance in physical sacred space (Dawson, 2005; O’Leary, 2005). As argued above, the body oriented, sensory enhanced, and experientially intense nature of the psychedelic experience renders the task of successfully translating it into online realm particularly daunting, given current technical limitations including latency, inferior video-audio fidelity and dependence on non-immersive screen interfaces. The impoverished, flat and pixelized qualities of online simulation become still more evident under the influence of psychedelics. My informants all agreed that online SD works are no substitute for on-site church-based rituals. Their responses included a variety of litanies about the constraints of virtual works. Compared with traditional SD rituals, virtual works discourage powerful inner voyages of transformation, present new challenges of distraction, and furthermore modify, erase and swaps diverse aspects of SD social and cultural context. In many respects, they represent a radically impoverished version of the ‘real thing.’ The more critical members of the informant pool were incisive in their judgment. They described virtual works as “a bumming experience” and “a way to connect that keeps us separate.”

Nevertheless, not all is negative. Some told of spiritual experiences had within *Live* rituals and asserted the spiritual authenticity of these works. Moreover, even the more critical voices conceded that virtual SD works had their place in the reality of social-isolation brought about by the COVID-19 pandemic. Pointing to the severity of mental health crisis experienced by Brazilian population in the wake of the pandemic (Goularte et al., 2021), one of my informants spoke of *Live* works as an antidote, allowing individuals to come together in a time of isolation. “It’s not a solution, but it’s something” told me another informant “my years of being a church member tell me that I need to work to maintain my connection to the SD.” For some church members, the realization of *Live* works represents an effort to not abandon one’s religion. Being able to join works online at a time when that wouldn’t otherwise had been possible allows the maintenance of the connection with one’s religious faith and practice.

*Live* works were generally seen as a fitting, *ad hoc* alternative for a moment of collective exigency. Asked whether Live works will continue in a post-pandemic world most of my informants suggested they probably will, but with less regularity, reserved for special occasions, courting to specific groups within the SD irmandade, and fulfilling a mostly social function. Asked whether they consider virtual SD rituals to be “real” works, they argued that online rituals perform a different function. Virtual works, in other words were viewed not as substitutive but rather as complementary, adding interconnection and complexification to SD practice (Sbardelotto, 2021). Importantly, while Campbell notes that some church members prefer online worship for reasons of conveniency and flexibility (Campbell, 2021a), none of my informants indicated anything of the sort, a fact that might be explained by the dramatic, spiritually intense, and scarcely reducible character of the psychedelic experience.

Finally, it is important to note that the *Live* genre of works still has a volatile, uncertain, plastic nature. Since August 2021, and as of the writing of these lines (February 2022) the organization of *Live* works by ICEFLU has ceased. Sources I have approached to inquire about the reason for this cessation have cited the more controlled COVID-19 situation in Brazil and the resumption of on-site works in Ceu do Mapiá. The demanding work required to organize multiple *Live* works was also cited. At the same time, non-ICEFLU online SD works are continuing, and ICEFLU organized works may resume in the future. Moreover, new more advanced technologies, such as virtual reality, might enter the picture in the future rendering the experience more immersive and attractive, and so the shape of online SD rituals is expected to further develop and evolve in the future.

## Conclusion

This paper contributes to the literature on the extra-pharmacological factors shaping experiences with psychedelics, as well as to the literature on the adoption of digital media technologies during the COVID-19 pandemic. It provides a unique perspective that combines these two fields of scholarship, focusing on the transformation of SD entheogenic rituals with the move online and the consequences for ritual participants.

This examination reveals both strengths and weaknesses. The migration online allowed for the continuation of SD entheogenic rituals at a time of social distancing and fostered a sense of global brotherhood. Concurrently, online ritual produced an impoverished ritual experience and novel types of challenges including a higher potential for distractions, technical difficulties, and low sensory fidelity. Other challenges included social anxiety and an in-built tension between the social and spiritual dimensions of ritual. Finally, some participants were concerned by the cultural context of online works: technological mediation, consumerism, commodification, and digital divide.

The study of online psychedelic rituals draws insights from the study of online religious rituals, the study of mediatization, and the turn online. Nevertheless, the intensified nature of the psychedelic experience (Hartogsohn, 2018; Timmermann et al., 2020) makes these rituals unique when compared with other online activities. Firstly, because of the intensified, sensitive nature of the psychedelic experience, these rituals are particularly high-stakes for participants. Moreover, the limitations of virtual space emerge all the more strikingly in the highly immersive, body oriented, experientially enhanced context of the psychedelic experience when compared with other instances of activities that migrated online (e.g., education, business, office work, non-psychedelic religious rituals). Current technology might be adequate to replace some types of everyday activities but judging from informants’ reports it has much to go before it is able to provide a viable alternative for embodied psychedelic rituals.

While this research is limited in scope and focused on the specific example of virtual SD rituals, it has implications for a wider range of issues and domains. Future research may pursue quantitative analyses of the types of shifts explored in this paper, expanding the number of surveyed individuals, as well as the number of groups and frameworks examined. Researchers may use the template of set and setting factors provided here to analyze other instances where entheogenic, and non-entheogenic religions are transformed by the migration online. Media ecology concepts like affordance may further serve to interrogate the ways of media environments reshape the characteristics of psychedelic experiences in virtual spaces, and religious experiences more generally. As growing parts of religious and entheogenic life move online, the questions what is gained? What is lost? And what possibilities lie ahead? Will continue to be resonate, prompting new investigations into novel forms of virtually mediated entheogenic ritual.

## Data Availability Statement

The datasets presented in this article are not readily available because of sensitive information which includes the illegal use of psychedelics. Requests to access the datasets should be directed to IH, idohartogsohn@gmail.com.

## Ethics Statement

The studies involving human participants were reviewed and approved by Bar Ilan University Interdisciplinary Studies Unit Ethics Committee. The patients/participants provided their written informed consent to participate in this study.

## Author Contributions

The author confirms being the sole contributor of this work and has approved it for publication.

## Conflict of Interest

The author declares that the research was conducted in the absence of any commercial or financial relationships that could be construed as a potential conflict of interest.

## Publisher’s Note

All claims expressed in this article are solely those of the authors and do not necessarily represent those of their affiliated organizations, or those of the publisher, the editors and the reviewers. Any product that may be evaluated in this article, or claim that may be made by its manufacturer, is not guaranteed or endorsed by the publisher.

## Footnotes

1 Set and setting is a key term in the field of psychedelic science, pointing to the crucial dependency of the psychedelic effects on contextual factors including expectancy, intention, physical, social and cultural environment (Hartogsohn, 2017).

2 See Dawson (2013 p. 35). The similarities and crucial differences between these lines are discussed extensively by Orgad (2012).

3 The term ‘Juramidam’ is a native SD term which denotes both God (Jura) and his soldiers (midam) thereby emphasizing the collective nature of SD work (Vasconcelos, 2017).

4 Due to the precarious legal situation of many SD communities, the specific location of churches is kept confidential.

5 In practice, few daimistas will realize such interest. My informants repeatedly pointed out that the live, in-time, quality of *Live* works is a key part of their attraction. Moreover, some of the *Live* works have been removed from the web following requests by organizing churches of particular works, or to because of errors in musical renditions of some hymns.

6 According to my sources these guidelines were introduced after some participants in early *Live* sessions appeared on screen involved in unrelated activities like “eating, talking or frying eggs.”

7 Importantly, the protocol fulfills a legal function too. Paragraphs from Brazil’s General Law of Data Protection (LGPD) are presented as part of the protocol in order to ensure that the work complies with privacy mandates specified by this law. It is interesting to think about the contextual difference of opening a spiritual work by reciting prayers, as commonly done in on-site SD works, and opening it by reading official legal code, as happens in *Live* works. Such legal considerations are notably also responsible for the proscription around drinking daime in front of the camera during *Live* works.

8 Importantly, several of my informants indicated that their commitment to virtual rituals was strengthened when joining in the company of friends, either physically co-present or even just virtually co-present in the event.

9 This might at first sight contradict with the above given description of on-site SD works as all-see-all affairs, where one is visible and potentially always observed. No contradiction exists, however. While members are encouraged to keep their eyes closed, some members may keep their eyes open, so that the participant is always potentially observed.

10 Importantly, this perspective, which was voiced by some Western interviewees, is quite different from that of Brazilians. Missionary trips and international tours have represented a major source of economic income and stability for some families of daimistas. This system of exchange was abruptly interrupted with the pandemic. Brazilians, particularly from Ceu do Mapia, therefore often view *Live* works as an opportunity to conduct campaigns to raise money for the support of their families and communities in the Amazon. See Viridi (2020).
